# The hidden benefits of helping students with systematic reviews

**DOI:** 10.5195/jmla.2018.420

**Published:** 2018-04-01

**Authors:** Rosie Hanneke

**Affiliations:** Assistant Professor and Information Services Librarian, Library of the Health Sciences–Chicago, University of Illinois at Chicago, 1750 West Polk Street, Chicago, IL 60612

## Abstract

Helping students with systematic reviews goes against the instinct of many librarians, who see it as their duty to talk researchers *out of* these projects rather than to assist them. My perspective on helping students with systematic reviews changed after meeting with one student a few years ago. However, the question of whether the finished product will be publication-worthy or entirely free of error is secondary, in my view, to other potential benefits to the student in completing the assignment.

My perspective on helping students with systematic reviews changed after meeting with one student a few years ago. As liaison librarian to the School of Public Health at the University of Illinois at Chicago (UIC), I meet with many master’s students who must conduct a research study as their capstone project before graduating. All capstone projects include a literature review component. Students may also choose to complete a stand-alone systematic review to fulfill the capstone requirement. A substantial number select this option, and several each year seek my guidance with the search.

While I had spoken with other students about systematic reviews before, generally to discourage them from attempting one, this was the first time a capstone student came to me and said that her advisor had directed her to conduct a systematic review—by herself and in a few months. “It can’t be done,” I told her, explaining that a systematic review requires a team of people and at least a year to complete. I suggested that she return to her advisor to develop Plan B, and I would be happy to assist her with the literature review component of whatever research project she decided on.

You may have guessed the ending of this story: I never heard from that student again. While I do not know how things turned out for her, I can imagine the strong likelihood that she continued on with her advisor’s original suggestion, only this time without a librarian to help guide her through the extensive literature search process.

Moving forward, I decided to change my perspective and approach these interactions in a more open, collaborative spirit. Rather than ending the conversation and essentially refusing help to a student who is taking on an overwhelming project, I decided to work with these students and help them as best I could. Once I changed my perspective, my eyes were opened to several potential benefits to students in conducting systematic reviews, as well as benefits to me as a librarian. I have developed an approach that works for me and that I hope will be informative to those of you who are interested in helping your own students.

## WHY SHOULD WE HELP?

Helping students with systematic reviews goes against the instinct of many librarians, who see it as their duty to talk researchers *out of* these projects rather than to assist them. It is arguably impossible for the average graduate student to complete a high-quality review in a single semester. However, the question of whether the finished product will be publication-worthy or entirely free of error is secondary, in my view, to other potential benefits to the student in completing the assignment.

Several authors have argued that conducting a systematic review can be beneficial to graduate students and residents. The systematic review is a relatively simple and inexpensive entry point for students to enter the scholarly conversation in their chosen disciplines under the guidance of a mentor [1]. It helps them become intimately familiar with the published literature, with a variety of research methods and statistical analyses [2], and with critical appraisal [3]. Importantly, the systematic review presents a way for students to make an intellectual contribution to their fields without requiring the resources that are often necessary for other types of research projects. Further, while we can imagine that many student reviews would not ultimately be of a quality suitable for publication, it is not inconceivable that a well-conducted review might result in a publication or conference presentation. In one case, the work completed by nineteen residents for a meta-analysis course ultimately resulted in eleven meeting abstracts and four publications, including two award winners [4].

From our own perspective, there are multiple possible benefits to librarians in assisting students with systematic review projects, at both the individual and course levels. One major advantage to us is the potential goldmine of insight into students’ searching skills and facility with bibliographic databases. Reviewing the detailed search strategies that students submit with their completed reviews can provide us with valuable information concerning the learning gaps that we might address with students [5]. This area of research has, so far, been under-explored by our profession.

Further, agreeing to meet with students individually or to provide information literacy instruction sessions for systematic review courses gives us a platform from which we can educate our users on best practices. If we do not take this opportunity, they are likely to be faced with completing the review assignment with few resources and without the guidance of a search expert. Simply put, by assisting these students, we support the populations that we serve. This includes individual students as well as faculty members, who often have compelling reasons for asking their students to complete systematic reviews.

## HOW CAN WE HELP?

When a student contacts me for assistance with a systematic review search, I schedule a sixty- to ninety-minute consultation appointment with them, at the library or at my office hours in the School of Public Health. I often send along a reading or two [6, 7] for the student to review in advance of our meeting. While the length and content of these consultations can vary greatly based on the student’s needs, experience, and progress on the review, we discuss the following at a minimum:

- the basic definition of a systematic review and what the process entails;
- the major guidelines for conducting and reporting systematic reviews, for example, the Cochrane Handbook and PRISMA;
- the need to search more than one database and which databases might be most suitable for their topics;
- the basics of searching (e.g., field tags, syntax) for at least one database;
- the importance of using both controlled vocabulary and keywords; and
- the organization and documentation of search strategy and references.

After this initial consultation appointment, students often contact me with questions via email. There are sometimes requests for a second appointment, usually when the student wants an in-depth walk-through of a citation manager. I have also conducted instruction sessions for a meta-analysis course, in which I addressed essentially the same topics, in greater detail.

## LESSONS LEARNED

In the brief time that I have been working with students in various stages of conducting systematic reviews, I have developed a few guiding principles that help me maintain sanity while still delivering quality support to the students.

First, ask the student if they hope to publish the systematic review or submit it to a conference. No matter their answer, and especially if they are working within an abbreviated timeframe, I clarify that there are typically stricter standards when publishing a systematic review than there are for the average student project. In my experience, most students are focused on completing the assignment at hand and have little to no desire to publish. However, a small but significant percentage of the students, often those in doctoral (PhD) programs, plan to submit the finished product to a conference or a journal. If these students are working with a strict assignment deadline, I advise that they will likely have to rework—if not completely redo—the review before it is of a publishable quality.

Second, make students aware of review types other than the systematic review. Sharing the article by Grant and Booth is particularly helpful [6]. Some faculty advisors may be stuck on the idea of students completing a “systematic review” and will consider no other type of review to be rigorous enough for the capstone project or thesis requirement (the label “rapid review” is often fitting but seems particularly stigmatized in this context). In other cases, the student is relieved to find another, more appropriate label to apply to their work. I have seen several instances where the student excitedly latched onto the concept of a scoping review, an apt characterization of their advisor’s vague direction to “see what’s been done on ______________.”

Finally, and perhaps most important to you as the librarian involved, set boundaries! Some students leave our first meeting feeling confident enough to complete the search on their own. Others, possibly overwhelmed or at least aware of their lack of knowledge in database searching, want me to be significantly more involved. I make every effort to be helpful while staying mindful of boundaries that I must place on my involvement in the project. When it comes to these student reviews, I am a liaison librarian, not a coauthor.

We must refrain from completing the student’s assignment for them, no matter how much our inner perfectionist wishes to swoop in to improve a less-than-ideal search. Students sometimes ask me to “check” their search strategies. In these cases, I scan the line-by-line search for errors in Boolean logic or search syntax. I do not check what I call, for lack of a better term, the *intellectual content* of the search strategy. For example, I do not investigate whether the student has included all Medical Subject Headings (MeSH) terms that are relevant to their review topics. I know that we discussed the importance of MeSH terms and how to find them in our consultation appointment, and it is, therefore, their responsibility to complete this task.

The student’s final search strategy is all but guaranteed to be of a poorer quality than one that you, an expert searcher, would complete yourself. That is okay. Step away from the search. Remember that the primary goal is a learning experience for the student.

In my view, this approach is not at odds with maintaining a high standard for ourselves as professionals. I consider it my duty to teach the gold standard for systematic review searching, no matter the individual circumstances of the researcher or the course. Students and faculty may decide to adapt these standards to their own resources and deadlines, applying them as they see fit. I think that many librarians feel that they must choose between helping students and their own professional integrity. In this situation, I do not believe that the two are mutually exclusive.

## MOVING FORWARD

At present, this approach works best for me. Since changing my perspective and working with the students and faculty, rather than against them, my eyes have opened to the positive aspects of what I formerly saw only as a negative. At the same time, I can see the downsides. Students are ill-prepared for and often overwhelmed by the project, one that their advisor may have unfortunately assured them would be “easy” or less time-consuming than another type of research study. Even when we are careful to set boundaries on our involvement in students’ reviews, this work is time-consuming for librarians and can pose a threat to maintaining a balanced workload [8].

My approach may change in the future. However, if I choose to seek change in this area, I plan to do so at the curricular level, for example, by perhaps encouraging faculty to rename the systematic review capstone option to “comprehensive” or “structured” literature review. Working with faculty to organize instruction efforts and ensure manageable systematic review search assignments can help us avoid being inundated with requests from students for individual support and consultation appointments [9]. This top-down approach seems more likely to effect real change, rather than denying students help at the individual level. I hope that we as a profession will continue to explore our students’ needs concerning systematic reviews as well as the ways that we can support these needs with integrity and understanding.
